# Supplementary material for: Audiovisual integration in the Mauthner cell enhances escape probability and reduces response latency
Source: Sci Rep. 2022 Jan 20;12:1097. doi: 10.1038/s41598-022-04998-2 (PMC8776867; doi:10.1038/s41598-022-04998-2)
Supplement: Supplementary file 1 — Supplementary Figures. [file 41598_2022_4998_MOESM1_ESM.pdf]

SUPP. FIGURE 1

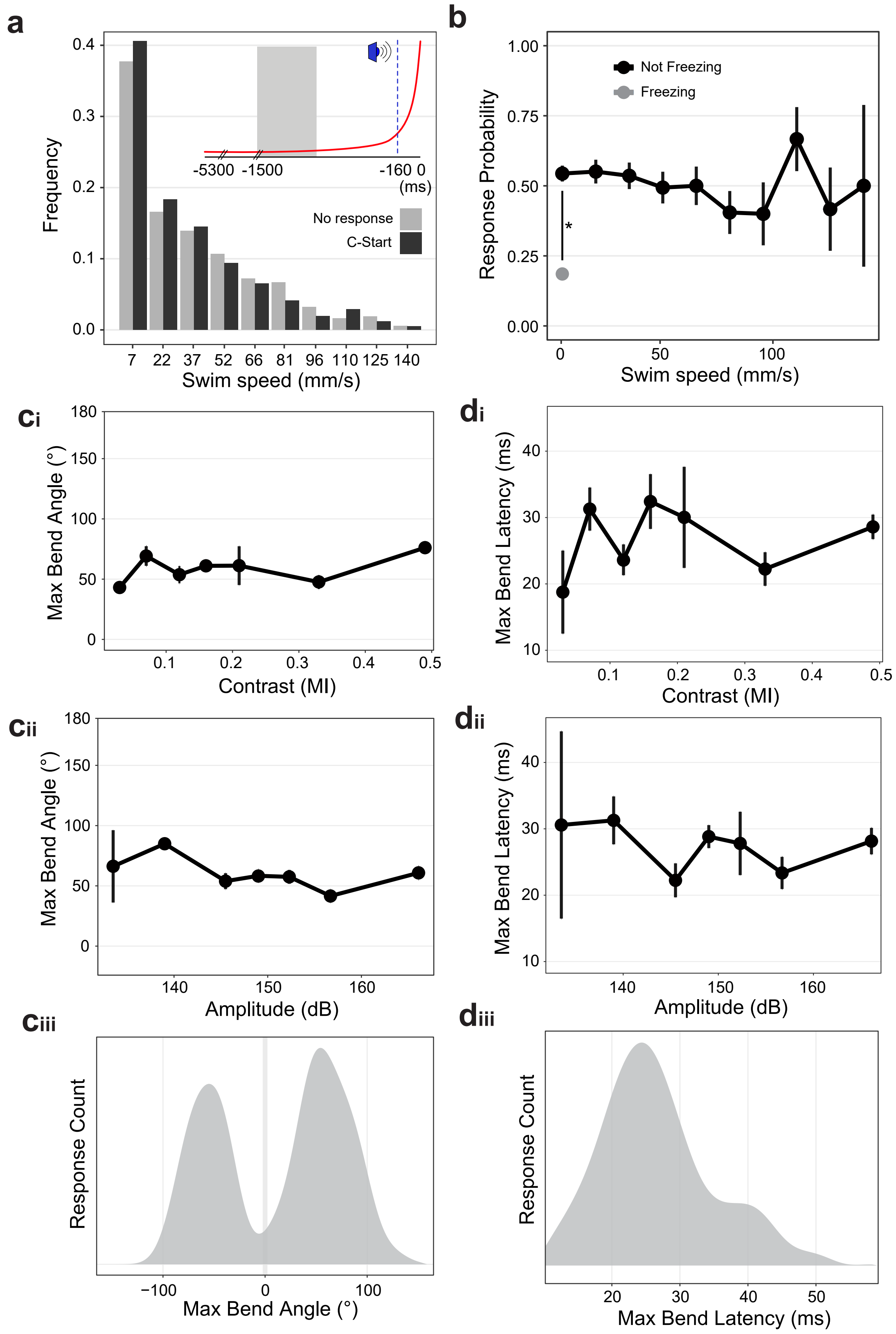

Supplementary Fig. S1. (**a**) Frequency distribution of swimming speeds prior to stimulation is similar in trials which ended in C-start or not (n C-start =414; n No-response =374; N=120). Inset shows the time interval (grey shading) where swim velocity was measured. (**b**) Response probability vs. swimming speed for non-freezing (black) compared to freezing animals (gray dot). Although C-start response probability does not depend on swimming speed, it is significantly higher for still (zero velocity) than truly freezing animals (binomial GLM, *p<0.001, n=727, N=125). (**c**) Maximum bend angle of the initial bend during C-starts in response to trials with a visual ($\mathbf{c}_{i}$) or auditory ($\mathbf{c}_{ii}$) component was not affected by intensity of the stimulus. The bimodal distribution of maximum bend angles ($\mathbf{c}_{iii}$) indicates that responses to the right or left of the animal had similar probability. (**d**) Duration of initial bend during C-starts in response to trials with a visual ($\mathbf{d}_{i}$) or auditory ($\mathbf{d}_{ii}$) component was not affected by intensity of the stimulus. Distribution of duration of the initial bend during the C-start peaked at about 25 ms ($\mathbf{d}_{iii}$). Data corresponds to a random subset of all trials including responses to unisensory or multisensory stimuli (12%, n=81/675, N=66).

SUPP. FIGURE 2

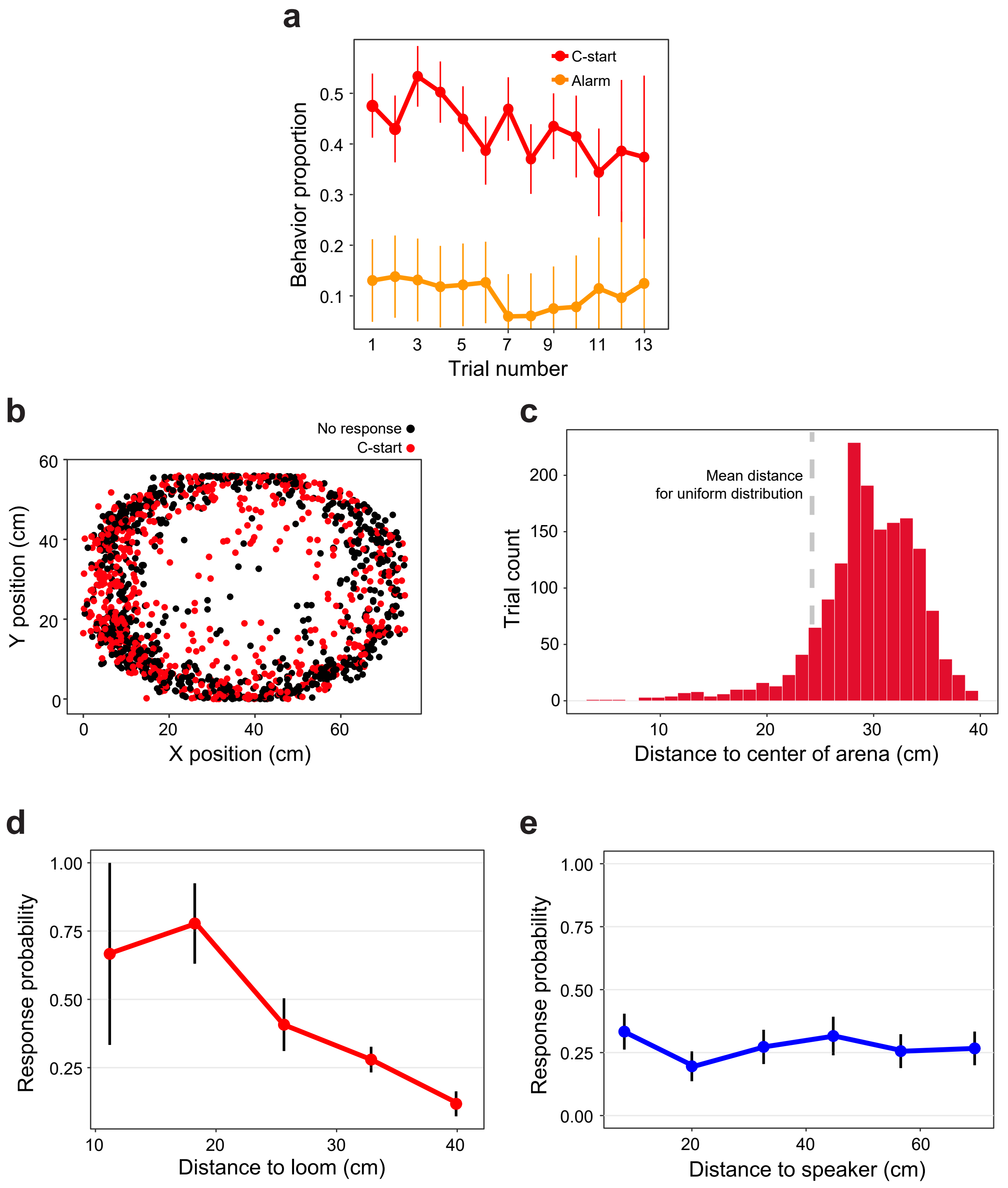

Supplementary Fig. S2. Influence of order of stimulus presentation and fish position in experimental arena. (**a**) Response probability decreases as the number of stimulations increases, especially for C-start responses but also slightly for alarm responses. Data from all behavioral experiments combined (n=1419, N=141). Error bars represent standard error for the proportion. (**b**) Position in experimental arena is not homogenous, a strong thigmotactic behavior is observed. Each dot represents the position of a fish in a single trial, at the time of C-start response (red dots), or 1500 ms before the end of visual expansion for non-responding animals (black dots). Data from all behavioral experiments combined. (**c**) Distance from fish head to the center of the experimental arena is not uniform. The distribution of distances is significantly shifted towards larger distances, confirming the thigmotactic behavior. Dotted gray line represents the mean of a theoretical uniform distribution. (**d**) Response probability decreases as distance to loom increases. Data from all unisensory visual stimuli combined (n=183, N=80). (**e**) Response probability is not dependent on distance to speaker. Data from all unisensory auditory stimuli combined. (n=261, N=106). In **a**, **d** and **e** black bars show binomial standard error.

SUPP. FIGURE 3

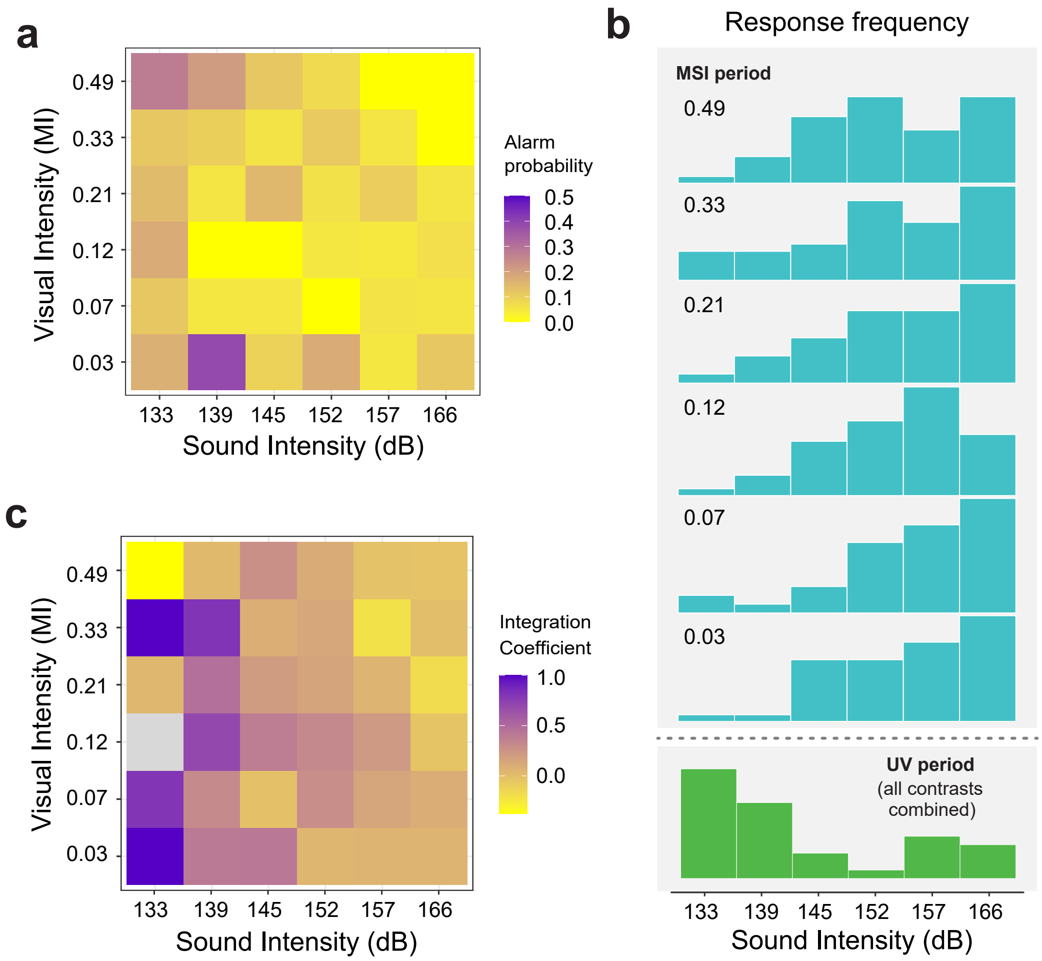

Supplementary Fig. S3. (**a**) Alarm response probability for each multisensory combination (binomial GLM, Alarm response ~ Intensity + Contrast, intensity: p=0.0006; contrast: p=0.967, n=665, N=88). Note the intensity scale runs from 0 to 0.5%. Since 93% of all alarm responses were performed by non-freezing animals, data was not split. (**b**) Histograms of the intensity of the sound component of responses on MSI period sorted by visual contrast (numbers in upper left corner, cyan) or during the UV period (green). In the latter, all contrasts where combined due to the low number of observations. (**c**) ICs during the MSI period for each combination of visual contrast and sound intensity. There were no responses for the combination shown in grey.
